# Diagnosing schizophrenia spectrum disorders: Large language models (LLMs) vs. leading international psychiatrists (LIPs)

**DOI:** 10.1111/pcn.13864

**Published:** 2025-07-05

**Authors:** Andrea Raballo, Federico Ravenda, Antonietta Mira

**Affiliations:** ^1^ REthinking MEntal Health Through Clinical and Data Intelligence (REMEDI) Lab, Faculty of Biomedical Sciences Euler Institute, Università della Svizzera Italiana Lugano Switzerland; ^2^ Cantonal Sociopsychiatric Organisation, Public Health Division, Department of Health and Social Care, Repubblica e Cantone Ticino Mendrisio Switzerland; ^3^ Department of Informatics Università della Svizzera Italiana Lugano Switzerland; ^4^ REthinking MEntal Health Through Clinical and Data Intelligence (REMEDI) Lab, Department of Economics Euler Institute, Università della Svizzera Italiana Lugano Switzerland; ^5^ Department of Science and High Technology University of Insubria Como Italy

The availability of large language models (LLMs) as putative diagnostic aids has a tangible transformative potential in many areas of science. Unlike many fields of medicine where diagnostic processes heavily rely on laboratory tests and imaging, psychiatric assessment fundamentally depends on narrative understanding and linguistic analysis of patients' experiences, emerging as relevant fields to further test LLMs potential for diagnostic purposes. A recent work by Li and colleagues^1^ provides the first comprehensive evaluation of LLMs in psychiatric assessment by testing GPT‐4, Bard, and Llama‐2 against both a standardized licensing examination and real clinical scenarios presented as multiple‐choice questions. Their study demonstrated that certain LLMs, particularly GPT‐4, could achieve diagnostic accuracy approaching that of expert psychiatrists.

Furthermore, a recent study by Urkin *et al*.^2^ demonstrated concerningly suboptimal levels of diagnostic accuracy among Leading International Psychiatrists (LIPs). Notably, when tasked with ascribing the best diagnostic estimate to two real‐world clinical vignettes of schizophrenia spectrum disorders (SSDs), only 33% correctly identified both test cases (an overt and a more sublet clinical presentation). The study, aimed at benchmarking diagnostic precision in psychiatry for SSD by quantifying the diagnostic performance of LIPs on simulated cases, raises critical questions about diagnostic reliability. Yet, it provides an opportunity to assess LLMs on the same diagnostic task, extending the work from Li *et al*. in a more challenging scenario where LLMs must generate diagnoses without predefined options, specifically focusing on SSDs, and using a broader range of LLMs, including both closed‐source and open‐source models.

For this reason, we tested multiple state‐of‐the‐art LLMs on the two vignettes presented in the original benchmark study^2^ (see: https://pmc.ncbi.nlm.nih.gov/articles/PMC11207759/). Among open‐source LLMs, we included large‐scale architectures (DeepSeek‐V3, Llama 3.1 405B, and Mixtral‐8\u00D722B) and lightweight ones (Phi‐3.5‐mini, Ministral‐8B, and Llama‐3.2‐3B). We also evaluated closed‐source LLMs including GPT‐4o, Claude‐3.5‐Haiku, and Gemini Flash 1.5. Each model was prompted in a Chain‐of‐Thought (CoT) fashion^3^ (i.e., prompting the model to reason step‐by‐step through symptoms and diagnostic criteria to reach a clinical conclusion, see Data S1 for the prompt template visualization) to provide diagnostic impressions. All models were used in inference mode by setting the temperature to zero to ensure that the outcomes (reported in the following Github repository: https://github.com/Fede-stack/LLMs-vs-LIPs) would be deterministic and thus reproducible. The experiments were conducted following TRIPOD‐AI research guidelines.^4^ No ethical approval was required for this study as it involved the use of anonymized clinical vignettes and computational analysis of publicly available large language models, without human participant involvement or access to personal data. The research adhered to established guidelines for artificial intelligence research (TRIPOD‐AI).

Figure 1 presents the results of the LLMs diagnostic evaluation of the two vignettes: closed‐source and large‐scale open‐source LLMs correctly identified the diagnosis for both vignettes, while open‐source lightweight LLMs showed selective competence, correctly diagnosing the first—most flamboyant SSD presentation—case, but misinterpreting the second—more attenuated SSD presentation—as various forms of depressive disorder.

**Fig. 1:**
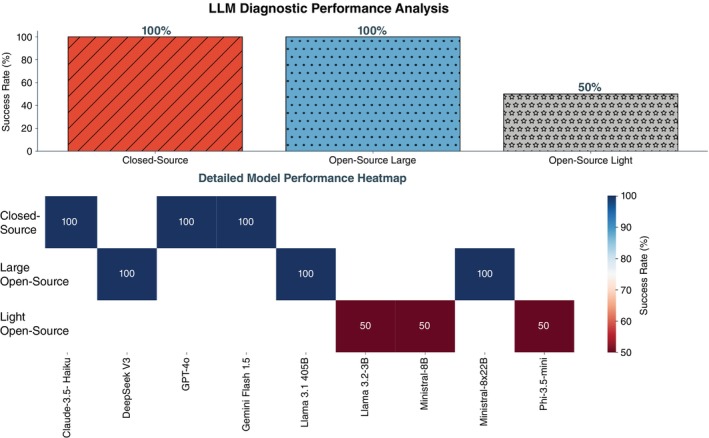
Diagnostic ascriptions given by different closed‐ and open‐source large language models (LLMs) on the two clinical vignettes previously used to benchmark leading international psychiatrists (LIPs) diagnostic accuracy. The barplot (up) shows the success rate by model category, while the heatmap (bottom) shows performance for each LLM considered.

While Li *et al*.^1^ demonstrated that earlier LLMs could approach psychiatrists' performance in structured multiple‐choice scenarios, our evaluation of more recent models shows that state‐of‐the‐art LLMs are comparable to the top‐performing 33% of diagnostically precise LIPs who correctly identified both SSD cases, without predefined diagnostic options. They effectively recognize both classical (e.g., David vignette) and subtle (e.g., Michael vignette) SSD presentations. These findings suggest that complex LLMs excel in psychiatric pattern recognition, particularly for nuanced clinical states, achieving expert‐level diagnostic precision on textual case material, and outperforming simpler LLMs models. This corroborates their potential as decision‐support tools to enhance diagnostic accuracy, reduce time‐to‐treatment in real‐world settings and marks a significant step toward clinically implementable AI assistance in psychiatric diagnosis.

Limitations: (i) For comparability reasons, the clinical testing material is limited to the two SSD vignettes previously used to benchmark LIPs diagnostic performance; (ii) we tested a subset of LLMs, although representative of current state of the art. (iii) While our focus was on diagnostic performance, we acknowledge that implementation of LLMs in psychiatric practice would require addressing considerations around potential algorithmic biases, privacy, and appropriate integration with clinical judgment. While broader testing incorporating a range of diverse clinical presentations is desirable to further validate LLMs performance across other diagnostic categories, the current study confirms their potential for diagnostic precision. Closed‐source and large‐scale open‐source LLMs achieve a level of accuracy comparable to that of the top‐performing LIPs, whereas lightweight LLMs, though suboptimal, still align with the performance of a substantial fraction of LIPs. Our findings highlight the potential of LLMs in psychiatric education and clinical support, especially in recognizing complex diagnostic patterns. Large‐scale training programs^5^ could benefit from AI‐guided decision support. Future research should investigate how LLMs might complement human expertise in psychiatric diagnosis while acknowledging the irreplaceable role of clinical experience and human judgment. Additionally, evaluating LLMs' diagnostic adaptability across different scenarios warrants further exploration.

## Disclosure statement

The authors declare no conflicts of interest, financial or otherwise, related to the content of this study.

## Supporting information


**Data S1.** Supporting information.

## Data Availability

These data were derived from the following resources available in the public domain: Github Repository and the data that support the findings of this study are available in Github Repository at https://github.com/Fede-stack/LLMs-vs-LIPs.
