# Supplementary material for: Diagnosing schizophrenia spectrum disorders: Large language models (LLMs) vs. leading international psychiatrists (LIPs)
Source: Psychiatry Clin Neurosci. 2025 Jul 5;79(9):599–600. doi: 10.1111/pcn.13864 (PMC12405820; doi:10.1111/pcn.13864)
Supplement: Supplementary file 1 — Data S1. Supporting information. [file PCN-79-599-s001.docx]

**Supplementary Material of:**

**Diagnosing Schizophrenia Spectrum Disorders: Large Language Models (LLMs) vs. Leading International Psychiatrists (LIPs)**

**Andrea Raballo,MD, PhD^1^, Federico Ravenda, Msc^3^, Antonietta Mira, PhD^4,5^**

^1^Faculty of Biomedical Sciences, Euler Institute, REthinking MEntal health through Clinical and Data Intelligence (REMEDI) Lab, Università della Svizzera Italiana, Lugano, Switzerland,

^2^Cantonal Sociopsychiatric Organisation, Public Health Division, Department of Health and Social Care, Repubblica e Cantone Ticino, Mendrisio, Switzerland.

^3^Department of Informatics, Università della Svizzera Italiana, Lugano, Switzerland.

^4^Department of Economics, Euler Institute, REthinking MEntal health through Clinical and Data Intelligence (REMEDI) Lab, Università della Svizzera Italiana, Lugano, Switzerland.

^5^Department of Science and High Technology, University of Insubria, Como, Italy

**Corresponding Author**

Corresponding Author:

Prof. Andrea Raballo, MD, PhD

Chair of Psychiatry, Faculty of Biomedical Sciences, University of Lugano, Lugano, Switzerland

andrea.raballo@usi.ch

**Figure 1 Supplementary.** Experimental workflow for evaluating LLM diagnostic capabilities using Chain-of-Thought prompting.

**Andrea Raballo,MD, PhD**

**Federico Ravenda, PhD student**

**Antonietta Mira, PhD**

**Author Affiliations**

Faculty of Biomedical Sciences, Euler Institute, REthinking MEntal health through Clinical and Data Intelligence (REMEDI) Lab, University of Southern Switzerland, Lugano, Switzerland; Cantonal Sociopsychiatric Organisation, Mendrisio, Switzerland (Raballo) Faculty of Informatics, Università della Svizzera italiana, USI, Lugano, Switzerland (Ravenda); Faculty of Economics, Euler Institute, REthinking MEntal health through Clinical and Data Intelligence (REMEDI) Lab, Università della Svizzera italiana, Lugano, Switzerland; Department of Science and High Technology, Insubria University, Como, Italy (Mira)

**Corresponding Author**

Corresponding Author:

Prof. Andrea Raballo, MD, PhD

Chair of Psychiatry, Faculty of Biomedical Sciences, University of Lugano, Lugano, Switzerland

andrea.raballo@usi.ch
